# Quantitative and Localization Fault Diagnosis Method of Rolling Bearing Based on Quantitative Mapping Model

**DOI:** 10.3390/e20070510

**Published:** 2018-07-06

**Authors:** Jialong Wang, Lingli Cui, Yonggang Xu

**Affiliations:** 1 Key Laboratory of Advanced Manufacturing Technology, Beijing University of Technology, Chaoyang District, Beijing 100124, China; 2 Beijing Engineering Research Center of Precision Measurement Technology and Instruments, Beijing University of Technology, Chaoyang District, Beijing 100124, China

**Keywords:** rolling bearing, quantitative and localization fault diagnosis, multiscale permutation entropy, multiscale morphological filtering, regression function

## Abstract

Aiming to solve the problem of accurate diagnosis of the size and location of rolling bearing faults, a novel quantitative and localization fault diagnosis method of the rolling bearing is proposed based on the quantitative mapping model (QMM). The fault size and location of the rolling bearing affect the impulse type and the modulation degree of the vibration signal, which subsequently changes the complexity and randomness of the time-domain distribution of the vibration signal. According to the relationship between the multiscale permutation entropy (MPE) of the vibration signal and rolling bearing fault size, an average MPE (A-MPE) index is proposed to establish linear and nonlinear QMMs through the regression function. The proper QMM is selected through the error rate of fault size prediction to achieve a quantitative fault diagnosis of the rolling bearing. Due to the mathematical characteristics of the QMM, the localization fault diagnosis is realized. The multiscale morphological filtering (MMF) method is also introduced to extract the time-domain geometric feature of the fault bearing vibration signal and to improve the QMM accuracy of the fault size prediction. The results show that the QMM has a great effect on the quantitative fault size prediction and localization diagnosis of the rolling bearing.

## 1. Introduction

Rolling bearing is an indispensable part of rotating machinery, but this can easily fail under harsh working conditions [1,2]. It is essential to detect the faults of rolling bearing, because the fault of rolling bearing can lead to the paralysis of machinery [3]. Bearing failure will not immediately lead to the collapse of rotating machinery, but as time shifts, the severity of the fault will increase, resulting in mechanical paralysis [4]. Fault prevention and restoration cannot get effective guidance if we only judge the fault degree of rolling bearings. The maintenance of equipment can be more effectively realized if the fault sizes and locations are accurately assessed.

There is a simple review of bearing fault diagnosis technology. The traditional fault diagnosis methods were mainly reflected in the fault feature extraction of vibration signals, such as wavelet transform (WT) [5], empirical mode decomposition (EMD) [6], etc. However, these methods only determined whether the bearing had a fault. Artificial intelligence (AI) has been introduced into bearing fault diagnosis in recent years [7]. AI is a powerful tool to improve the efficiency of fault diagnosis [8]. The results of intelligent diagnosis were obtained by artificial neural networks (ANN) [9,10] and fuzzy logic [11]. These AI methods had realized the classification of bearing faults. Wang et al. [12] proposed the method to identify compound faults of bearing based on ensemble empirical mode decomposition (EEMD) and independent component analysis (ICA) method; results showed that the proposed method is effective in compound fault separation. Although these studies have realized bearing fault classification and bearing compound fault separation, the research on the quantitative analysis of bearing fault is insufficient.

Due to insufficient research on the quantitative diagnosis of rolling bearing faults, a new double-dictionary matching pursuit (DDMP) method based on the Lempel-Ziv complexity (LZC) index is proposed to analyze the damage degree of bearings [13]. Cui et al. [14] discussed the fault mechanisms of the rolling bearing outer ring and proposed a quantitative and localization diagnosis method based on the vertical–horizontal synchronized root mean square (VHSRMS) index to quantitatively analyze rolling bearings. These articles did not predict the specific fault size of rolling bearings. Zhao et al. [15] combined EMD and approximate entropy, before selecting IMF through an approximate entropy. The obtained IMF was analyzed to calculate the fault size of bearings but it is difficult to determine the fault size under heavy noise. In order to solve these problems, the quantitative mapping model (QMM) based on the average multiscale permutation entropy (A-MPE) of the vibration signal and the fault size of bearing is established in this paper, before the prediction of bearing fault size and localization diagnosis are realized. The fault size is predicted by the mapping relation. The permutation entropy (PE) algorithm proposed by Bandt and Pompe [16] is a method of detecting the random and dynamic mutation of time series, which has the characteristics of simple calculation and strong robustness. The fault size and location of the rolling bearing affect the impulse type and the modulation degree of the vibration signal. With the increase of bearing outer ring faulty size, the rising edge of the pulse is generated when the rolling element just gets in touch with the fault edge, and the falling edge of the pulse is generated when the rolling element leaves the other edge of the defect, which causes a double impact for the vibration signal. In this case, the complexity and randomness of the vibration signal is enhanced, which leads to the increase of multiscale permutation entropy (MPE). With the increase of bearing inner ring fault size, vibration signal will have a phenomenon of low-frequency amplitude modulation, which will reduce the complexity and randomness of the vibration signal, and then MPE will be reduced. According to the above analysis, MPE can be used as an index for quantitative analysis of bearing faults.

Some scholars have introduced PE for bearing fault detection and achieved good results. Yan et al. [17] compared the PE with the Lempel–Ziv complexity and the results show that PE can detect and amplify the dynamic changes of vibration signal more effectively. Wei et al. [18] analyzed the vibration signal of a gearbox by the local mean decomposition (LMD) method and evaluated the PE of the obtained product function (PF). According to different PEs, the fault identification of the gear box is realized. It is difficult to extract the weak fault feature information only using single scale analysis, since the vibration signal of the mechanical system contains rich feature information and it is necessary to analyze the vibration signal at multiple scales [19]. Vakharia et al. [20] used MPE as an indicator of characteristic information to select the best wavelet, before the fault diagnosis of rolling bearing was realized. With the development of science and technology, the MPE and AI are applied to the bearing fault diagnosis, and the bearing fault is classified. Li et al. [21] proposed a new rolling bearing fault diagnosis method based on MPE and the improved support vector machine (SVM) based binary tree (ISVM-BT) to realize automatic fault identification. Zheng et al. [22] applied generalized composite MPE and Laplacian score to diagnose faulty bearings, with the results showing that this algorithm greatly improves the fault recognition rate. The authors of Reference [23] combined MPE with SVM and proposed a new rolling bearing fault diagnosis strategy to judge the location of rolling bearing faults. Jiang et al. [24] improved the MPE and proposed a new intelligent fault diagnosis model based on multiscale weighted permutation entropy (MWPE) and extreme learning machinery (ELM) to realize the fault identification of bearings and gears.

Although some studies have applied MPE for the fault diagnosis of rolling bearings, most of these studies conducted a qualitative analysis. As such, MPE has not yet been introduced into the quantitative fault diagnosis of rolling bearings. In order to map the relationship one by one, the average MPE (A-MPE) is proposed to realize the quantitative prediction of bearing fault sizes in this paper.

The vibration signal of faulty bearings collected from the experimental setup is often accompanied by heavy noise and interference signals, which directly affect the actual complexity of the vibration signal and subsequently affect the fault prediction accuracy. In order to better extract the impulse characteristics of bearing vibration signals under different fault sizes, the multiscale morphological filtering (MMF) method was introduced into this paper. The morphological filtering (MF) method directly extracts the geometric features in the time domain and can effectively extract the fault feature information [25]. The main intention of the MMF method is to select a structural element (SE) for morphological operations, which finally eliminates noise and extracts the information of impulse characteristics [26]. Tan et al. [27] proposed an optimal MMF method to extract the feature information of faulty rolling bearing vibration signals. Zhang et al. [28] applied the MMF method to bearing fault diagnosis and the results showed that multiscale morphological analysis is very effective in extracting the morphological characteristics of the vibration signals. In this paper, the faulty bearing vibration signal is analyzed by the MMF method to extract the time-domain geometric feature, before solving the A-MPE of the analyzed vibration signal, which greatly improves the accuracy of the QMM in the quantitative prediction of bearing fault size.

The rest of this paper is structured as follows: Section 2 mainly introduces the mathematical morphological filter (MMF). Section 3 introduces the average multiscale permutation entropy (A-MPE) index proposed in this paper. In Section 4, the quantitative mapping model (QMM) is proposed. Section 5 verifies that the QMM method is applied to the experimental signal to predict fault size and location. The conclusions are drawn in Section 6.

## 2. Mathematical Morphological Filter (MMF)

### 2.1. Morphological Filtering Operators

Morphological filter (MF) plays an important role in the field of nonlinear signal processing. There are many basic operations of MF, including dilation, erosion, opening and closing [29].

We assumed that the definition domain of the signal *f*(*n*) is *F* = (0, 1, ..., *N* − 1) and the definition domain of the signal *g*(*n*) is *G* = (0, 1, ..., *M* − 1), where *N* and *M* are the number of sampling points, and *N* ≥ *M*. *f*(*n*) is the vibration signal to be analyzed, and *g*(*n*) is the structural element (SE). The dilation and erosion of *f*(*n*) by *g*(*n*) is written as:

(1) $$\begin{array}{l} f⊕g(n)=\max[f(n-m)+g(m)]\\ \;m\in 0,1,2......M-1 \end{array}$$

(2) $$\begin{array}{l} f⊖g(n)=\min[f(n+m)-g(m)]\\ \;m\in 0,1,2......M-1 \end{array}$$

The opening and closing of *f*(*n*) by *g*(*n*) is defined as:

(3) (f∘g)(n)=(f⊖g⊕g)(n)

(4) (f•g)(n)=(f⊕g⊖g)(n)

where symbols ⊕ and ⊖ represent respectively dilation and erosion operations, symbols ∘ and • represent respectively opening and closing operations.

Top-hat transform and top-hat’s dual operator are written as:

(5) HAT(f)=f•g(n)−f

(6) HAT(−f)=f−f•g(n)

The difference operator and gradient operator of *f*(*n*) by *g*(*m*) are defined as:

(7) $$f_{DIF}(n)=f•g(n)-f\circ g(n)$$

(8) $$f_{AGV}(f)=f⊕g(n)-f⊖g(n)$$

The multiscale analysis is applied to signal processing in order to obtain a better filter. Let *g*(*n*) be the SE and *ε* (*ε* = 1, 2, ..., *λ*) be the scale. The multiscale dilation and erosion can be written as:

(9) $$(f⊕\varepsilon g)(n)=\underset{(\lambda -1)Time}{\underset{︸}{f⊕g⊕g\cdot \cdot \cdot g(n)}}$$

(10) $$(f⊖\varepsilon g)(n)=\underset{(\lambda -1)Time}{\underset{︸}{f⊖g⊖g\cdot \cdot \cdot g(n)}}$$

The multiscale difference filter can be expressed as:

(11) $$y_{\varepsilon }(n)=(f•\varepsilon g)(n)-(f\circ \varepsilon g)(n)$$

**Figure 1:**
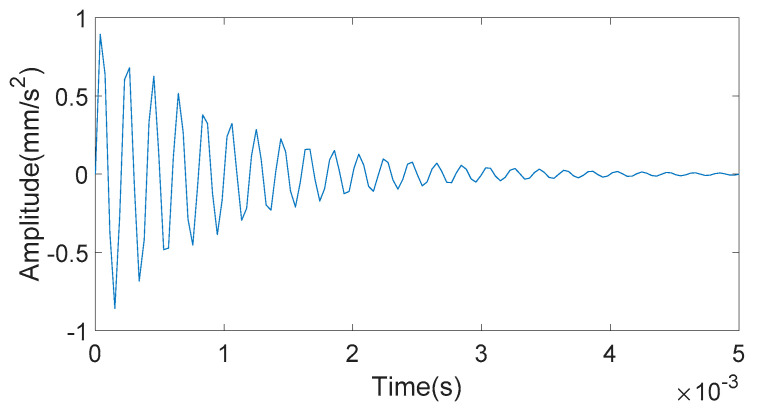
Exponential decay signal.

In order to obtain the filtering effect of different morphological operators, an exponential decay signal is constructed, which is shown in Figure 1.

**Figure 2:**
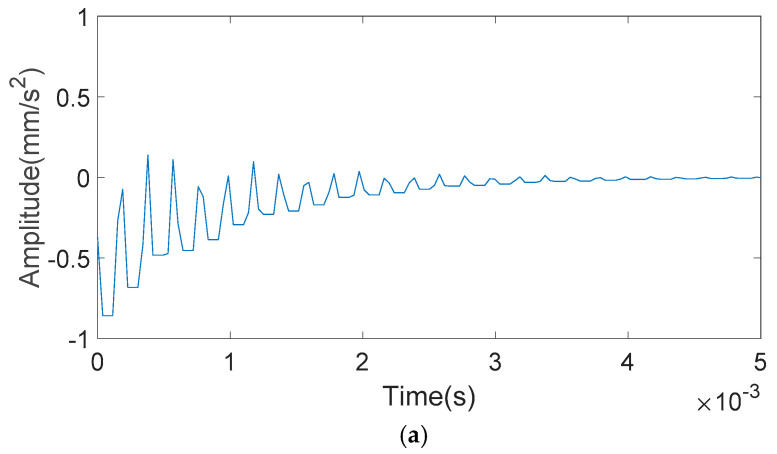

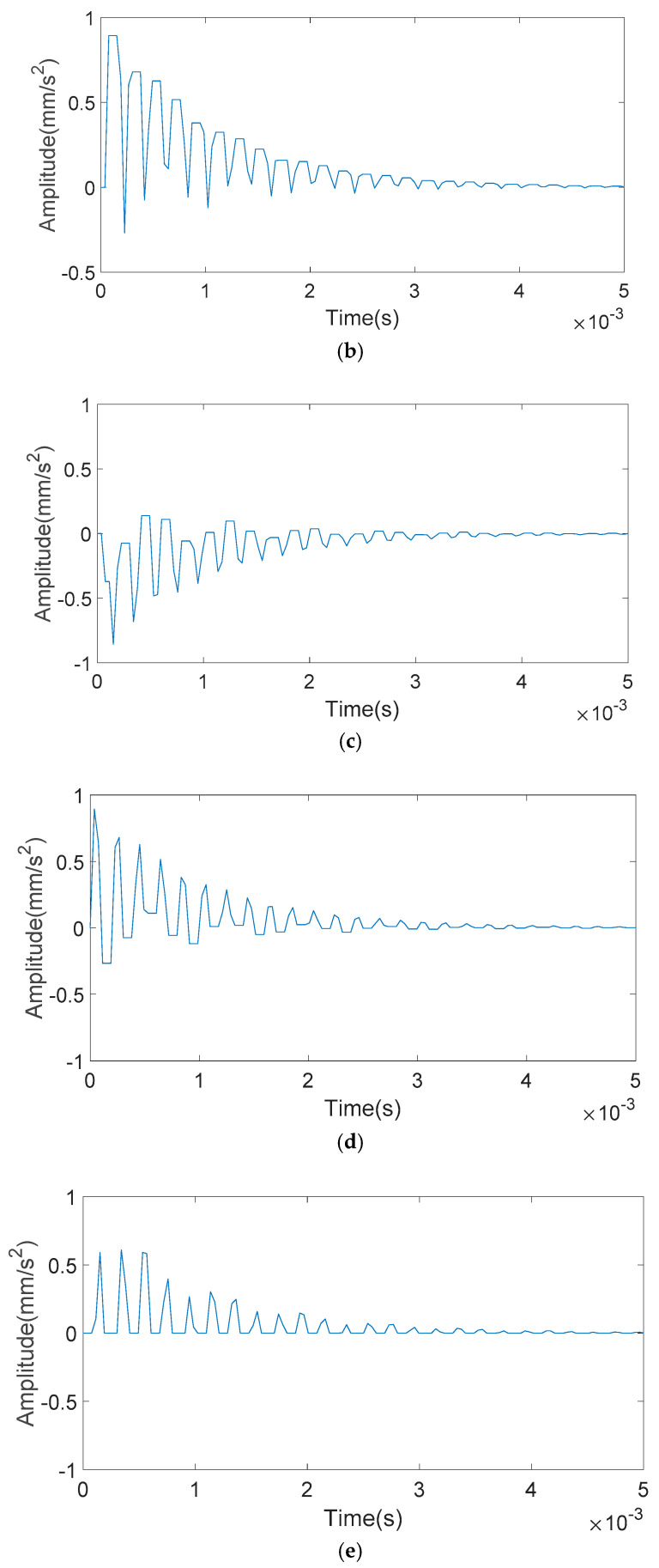

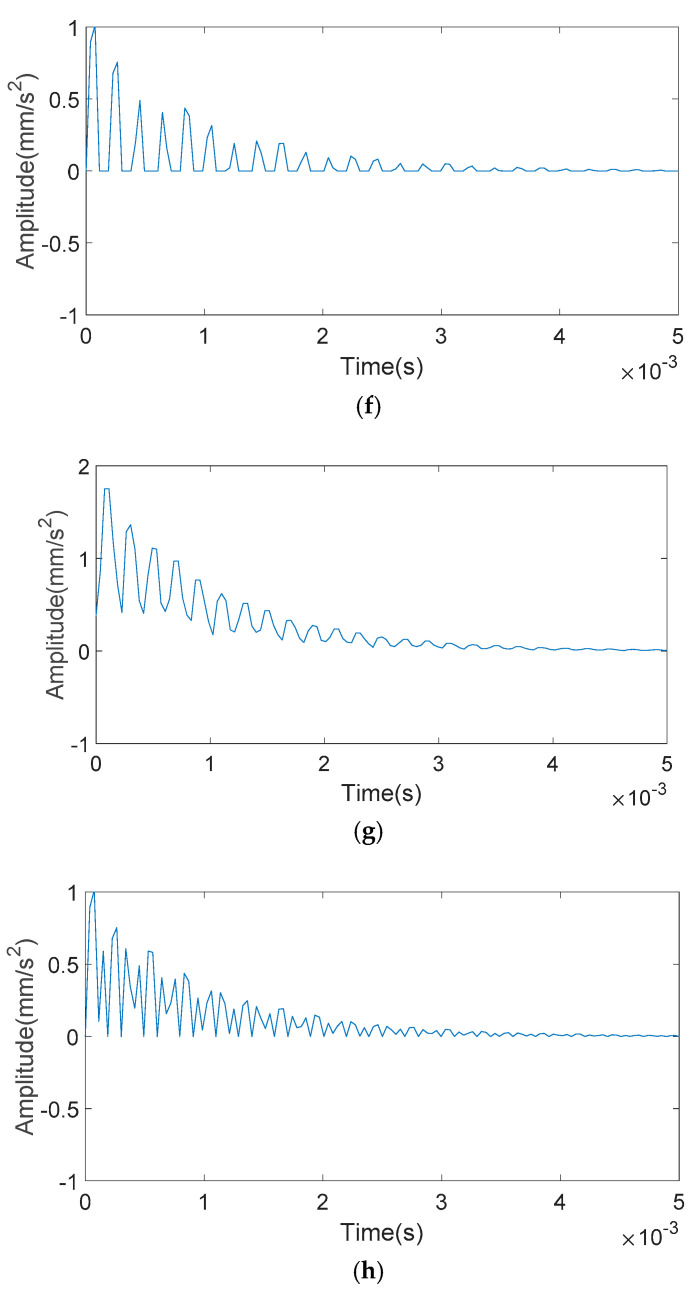
Morphological operations of the exponential decay signal: (**a**) Erosion operator; (**b**) dilation operator; (**c**) opening operator; (**d**) closing operator; (**e**)top-hat operator; (**f**) top-hat’s dual operator; (**g**) gradient operator; and (**h**) difference operator.

The exponential decay signal is morphologically analyzed by different morphological operators, which is shown in Figure 2. The filtering effects of morphological operators for the positive and negative impact are shown in Table 1.

It is clearly seen in Table 1 that the opening operation plays a smooth role on the positive impulse of a one-dimensional signal. The effect of the closing operation is exactly the opposite of the opening operation. The difference filter that includes the opening and closing operator can better extract the positive and negative impulses in the vibration signal.

### 2.2. Structure Element (SE)

Apart from the combination of morphological operators, the structural element (SE) also plays a decisive role in signal processing. SEs are determined by the shape and scale. There are many general shapes of the SEs, including flat SE, semicircle SE and other multiple curves. The flat SE is the simplest SE, and has been proved to be effective in bearing fault detection [30,31,32,33]. In order to reduce the running time of CPU, flat SE is used to morphologically analyze the vibration signals in this paper. The relationship between the length and scale of flat SE is shown in Table 2, where height determines shape of SE, and there is a certain relationship between the scale and the length.

For optimal scale, Raj et al. [34] proposed an optimal scale selection method of multiscale morphological filtering using the kurtosis principle (KS-MMF) and the method can be described as follows: The original vibration signal was first morphologically analyzed with SEs of 10 different scales spaced by 10% of the pulse duplicate period. After this, the kurtosis of the morphologically filtered signals was calculated. The filtered signal with the maximum kurtosis value is selected for further analysis.

## 3. Average Multiscale Permutation Entropy (A-MPE)

After reconstructing the phase space for the vibration signal sequence {*x*(*i*), *i* = 1, 2, ..., *N*}, where *N* is the sampling point, we obtained the following sequence:

(12) $$\left\{\begin{array}{c} X(1)=\{x(1),x(1+\lambda ),\cdot \cdot \cdot \cdot \cdot \cdot ,x(1+(m-1)\lambda )\}\\ .......\\ .......\\ X(i)=\{x(i),x(i+\lambda ),\cdot \cdot \cdot \cdot \cdot \cdot ,x(i+(m-1)\lambda )\}\\ .......\\ .......\\ X(N-(m-1))=\{x(N-(m-1)\lambda ),x(N-(m-2)\lambda ),\cdot \cdot \cdot \cdot \cdot \cdot x(N)\} \end{array}\right\}$$

where *m* is the embedding dimension and *λ* is the time delay.

*X*(*i*) can be rearranged in ascending order as:

(13) $$X(i)=\left\{x(i+(j_{i1}-1)\lambda )\leq x(i+(j_{i2}-1)\lambda )......\leq x(i+(j_{m}-1)\lambda )\right\}$$

If $x(i+(j_{i1}-1)\lambda )=x(i+(j_{i2}-1)\lambda )$ exists, it will be newly arranged according to *j*, which produces namely $x(i+(j_{i1}-1)\lambda )\leq x(i+(j_{i2}-1)\lambda )$. Therefore, any data *X(i)* can get a set of symbols.

(14) $$S(g)=\left\{j_{1},j_{2},.....j_{k}\right\}$$

where *g* = 1, 2, 3, ..., *k*, k≤m!; *m*! is the largest number of different symbols and *S*(*g*) is one of *m* different symbols.

We calculated the occurrence probability of each symbol *S*(*g*) and $\sum_{g=1}^{k}P_{g}=1$. The Permutation Entropy (PE) is described as follows:

(15) $$H_{P}(m)=-\sum_{g=1}^{k}P_{g}\ln P_{g}$$

where *H_P_*(*m*) is PE value, and *P_g_* is the probability of each symbol *S*(*g*). 

When the PE is standardized, we obtain the following:

(16) $$H_{P}=H_{P}(m)/\ln(m!)$$

where *m* is the embedding dimension, and *H_P_* is normalized *PE* value.

The multiscale permutation entropy (MPE) is defined as the PEs under different scales and the calculation method is as follows.

The vibration signal sequence {*x*(*i*),*i* = 1, 2, ..., *N*} with length *N* is refined, which obtains the sequence {*y^s^_j_*}. *y^s^_j_* and can be described as:

(17) $$y_{j}^{s}=\frac{1}{s}\sum_{i=(j-1)s+1}^{js}x_{i},(s=1,2,.........[N/s])$$

where *s* is the scale factor.

We calculated the PEs of the vibration signal sequence under different scales and solve the mean value of PEs. The average multiscale permutation entropy (A-MPE) can be described as:

(18) $$Ave(H_{P})=\frac{\sum_{l=1}^{s}H_{P}(l)}{s}$$

where *Ave* (*H_P_*) is average multiscale permutation entropy, *H_P_*(*l*) is PE value, and *s* is the scale factor.

Obviously, the range value of *Ave*(*H_p_*) is $0\leq Ave(H_{P})\leq 1$. *Ave*(*H_p_*) represents the complexity of the signal sequence. A larger *Ave*(*H_p_*) indicates a more complex time series. Conversely, the time series is more regular. The *Ave*(*H_p_*) magnifies the local subtle change in the time series. With a change in rolling bearing fault size, the complexity of the vibration signal will change, which will change *Ave*(*H_p_*).

## 4. Quantitative Mapping Model (QMM)

### 4.1. Establishment of QMM

The QMMs established in this paper include the linear model and nonlinear model.

(1) The linear regression model is used to construct linear QMM. The linear regression model is introduced as follows:

We took *N* different values {*x*_1_, *x*_2_, ..., *x_n_*} in the discrete sequence *x* for independent testing, which can obtain *N* samples {(*x*_1_, *y*_1_), (*x*_2_, *y*_2_), ..., (*x_n_*, *y_n_*)}. The linear regression equation can be expressed by Equation (19):

(19) $$y_{i}=a+bx_{i}+\varepsilon_{i},\;\varepsilon_{i}\sim N(0,\sigma^{2})$$

where *a* and *b* are pending parameters, *ε_i_* are mutually independent, *i* = 1, 2, ..., *n*, *ε_i_* conforms to normal distribution, and *σ*^2^ is variance.

The joint density function of {*y*_1_, *y*_2_, ..., *y_n_*} is shown in the following Equation:

(20) $$L={\left(\frac{1}{\sigma \sqrt{2\pi }}\right)}^{n}\exp{\left[-\frac{1}{2\sigma^{2}}\sum_{i=1}^{n}(y_{i}-a-bx_{i}\right)}^{2}]$$

where *L* is joint density function, *a* and *b* are pending parameters.

The maximum likelihood estimate (MLS) method is used to estimate the unknown parameters *a* and *b*. Equation (20) is the likelihood function of the sample. Obviously, *L* is required to have a maximum value. In other words, Equation (21) needs to have a minimum value:

(21) $$Q(a,b)=\sum_{i=1}^{n}{\left(y_{i}-a-bx_{i}\right)}^{2}$$

where *Q*(*a*,*b*) is the likelihood function of the sample, *a* and *b* are pending parameters. 

We solved the partial derivative of *Q* by letting the partial derivative be 0:

(22) $$\begin{array}{l} \frac{\partial Q}{\partial a}=-2\sum_{i=1}^{n}(y_{i}-a-bx_{i})=0\\ \frac{\partial Q}{\partial b}=-2\sum_{i=1}^{n}(y_{i}-a-bx_{i})x_{i}=0 \end{array}\}$$

According to Equation (23), the MLS values of *a* and *b* are obtained:

(23) $$\begin{array}{l} \hat{b}=\frac{\sum_{i=1}^{n}\left(x_{i}-\frac{1}{n}\sum_{i=1}^{n}x_{i})(y_{i}-\frac{1}{n}\sum_{i=1}^{n}y_{i}\right)}{\sum_{i=1}^{n}{\left(x_{i}-\frac{1}{n}\sum_{i=1}^{n}x_{i}\right)}^{2}}\\ \hat{a}=\frac{1}{n}\sum_{i=1}^{n}y_{i}-\hat{b}\;*\frac{1}{n}\sum_{i=1}^{n}x_{i} \end{array}\}$$

where $\hat{a}$ and $\hat{b}$ represent respectively MLS values of *a* and *b*.

The MLS values of *a* and *b* are substituted into Equation (19), which can obtain the regression function.

(2) The nonlinear regression model is used to construct the nonlinear QMM. The nonlinear regression model is introduced as follows.

We took *N* different values {*x*_1_, *x*_2_, ..., *x_n_*} in the discrete sequence *x* for independent testing, which can obtain *N* samples {(*x*_1_, *y*_1_), (*x*_2_, *y*_2_), ..., (*x_n_*, *y_n_*)}. The fitting polynomial is shown by the following Equation:

(24) $$y=a_{0}+a_{1}x+......+a_{n}x^{n}$$

where *a*_0_, *a*_1_, ..., *a_n_* are pending parameters.

The sum of the distances between the sample points {(*x*_1_, *y*_1_), (*x*_2_, *y*_2_), ..., (*x_n_*, *y_n_*)} and the fitting polynomial is the sum of the square of the deviations (DEVSQ).

(25) $$R^{2}=\sum_{i=1}^{n}{\left[y_{i}-(a_{0}+a_{1}x+......+a_{n}x^{n})\right]}^{2}$$

where *R*^2^ is the fitting polynomial.

In order to obtain each parameter, we solved the partial derivative of each parameter for Equation (24), which gives the following Equation:

(26) $$\begin{array}{l} -2\sum_{i=1}^{n}[y_{i}-(a_{0}+a_{1}x+....+a_{n}x^{n})]=0\\ -2\sum_{i=1}^{n}[y_{i}-(a_{0}+a_{1}x+....+a_{n}x^{n})]x=0\\ \;........\\ -2\sum_{i=1}^{n}[y_{i}-(a_{0}+a_{1}x+....+a_{n}x^{n})]x^{n}=0 \end{array}\}$$

Equation (26) is converted to a matrix and the following matrix is obtained:

(27) $$\left[\begin{array}{l} n\;\sum_{i=1}^{n}x_{i}\;...\;\sum_{i=1}^{n}x_{i}{}^{n}\\ \sum_{i=1}^{n}x_{i}\;\sum_{i=1}^{n}x_{i}^{2}\;...\;\sum_{i=1}^{n}x_{i}{}^{n+1}\\ \;......\;\\ \sum_{i=1}^{n}x_{i}{}^{n}\;\sum_{i=1}^{n}x_{i}^{n+1}\;...\;\sum_{i=1}^{n}x_{i}{}^{2n} \end{array}\right]\;\left[\begin{array}{l} a_{0}\\ a_{1}\\ ....\\ a_{n} \end{array}\right]=\left[\begin{array}{l} \sum_{i=1}^{n}y_{i}\\ \sum_{i=1}^{n}x_{i}y_{i}\\ \;....\\ \sum_{i=1}^{n}x_{i}^{n}y_{i} \end{array}\right]$$

Equation (27) is written as X*A = *Y*. The *A* matrix is solved by the matrix operation and the parameter values are obtained. The parameter values are substituted into Equation (24), which can obtain a nonlinear regression function.

### 4.2. The Steps of Establishing QMM

The steps of the quantitative and localization fault diagnosis method for rolling bearings based on QMM proposed in this paper are as follows:

(1) Collecting the vibration signals of rolling bearings under different fault sizes of the outer and inner rings.
(2) The vibration signals of different fault sizes are analyzed morphologically (Equation (11) and the preprocessed signals are obtained.
(3) Solving the A-MPEs of the preprocessed signals.
(4) Fitting the A-MPEs through a regression model (Equations (19) and (24)) to obtain linear and nonlinear QMMs.
(5) According to the mathematical characteristics of the QMM, the fault localization diagnosis is realized.
(6) In order to verify accuracy, linear and nonlinear QMMs are used to predict the fault size of rolling bearings and to select the appropriate QMM through the rate of errors in predicting the fault size.

**Figure 3:**
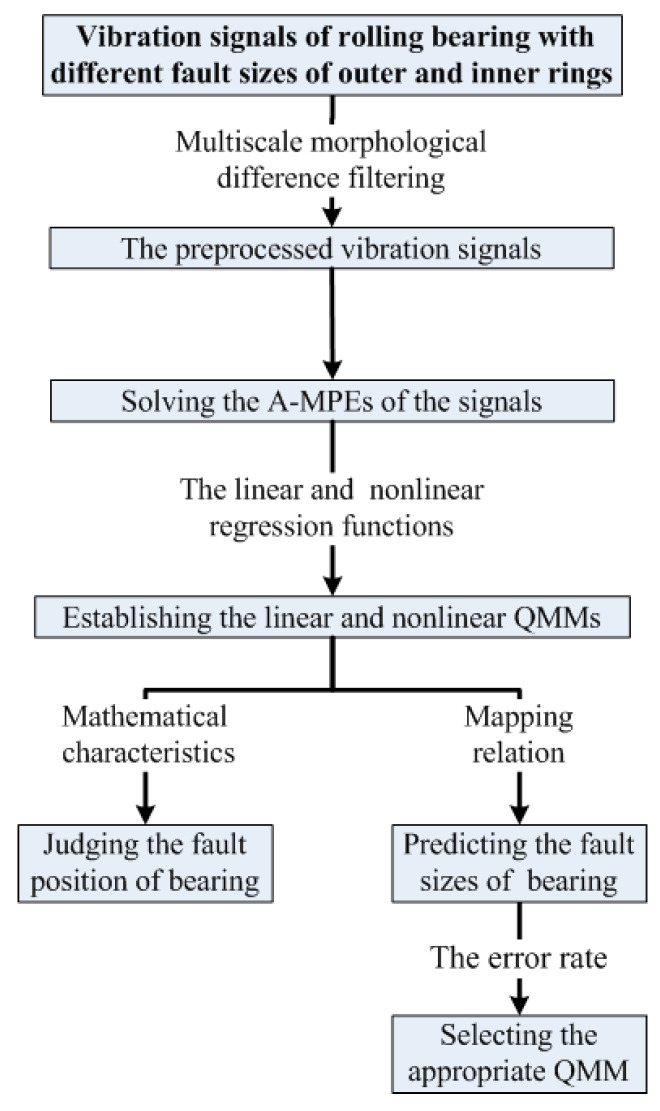
Flowchart of the QMM method.

The diagram of the QMM method is shown in Figure 3.

## 5. Experimental Verification

### 5.1. Vibration Signal of Rolling Bearing

**Figure 4:**
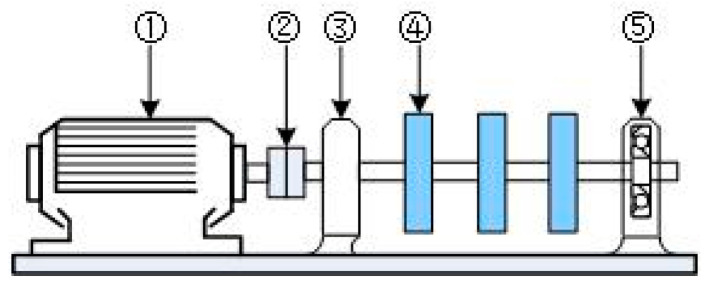
The experimental setup.

**Figure 5:**
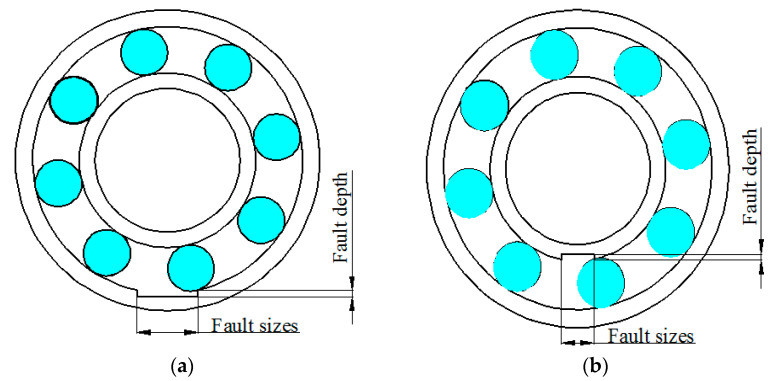
Sketch of faulty bearing: (**a**) Outer race fault; and (**b**) Inner race fault.

The experimental system consists of a bearing experimental stand, a data collection instrument of HG3528A and a laptop computer. The experimental setup is shown in Figure 4. The three-phase asynchronous motor ① is connected to the shaft equipped with a rotor ④ through the flexible shaft coupling ②. The shaft is supported by the two 6307 bearings: ③ is the normal bearing, while ⑤ is the bearing with different fault sizes. The bearings have an outer diameter D = 80 mm, inner diameter d = 35 mm, 8 rolling elements (Z) and contact angle α = 0. The motor speed (R) was 1497 r/min. Rolling bearings with different fault sizes and same fault depths are produced by electro-sparking manually. A sketch of the fault bearing is shown in Figure 5. The shape of the fault is rectangular, and the fault parameter is “size×depth×width”. The fault sizes are 0.5 mm, 2 mm, 3.5 mm and 5 mm separately. The fault depth is 0.1 mm, and the fault width is 21 mm (bearing thickness). Experimental data of the vibration signal under the condition of each fault size are obtained through a PCB-601A02 acceleration sensor installed in the radial direction of the bearing block of the faulty bearing. The sampling frequency was set to 12,800 Hz, and the number of sampling points *n* = 8192. In order to improve the computational efficiency, we choose 1800 points for analysis.

**Figure 6:**
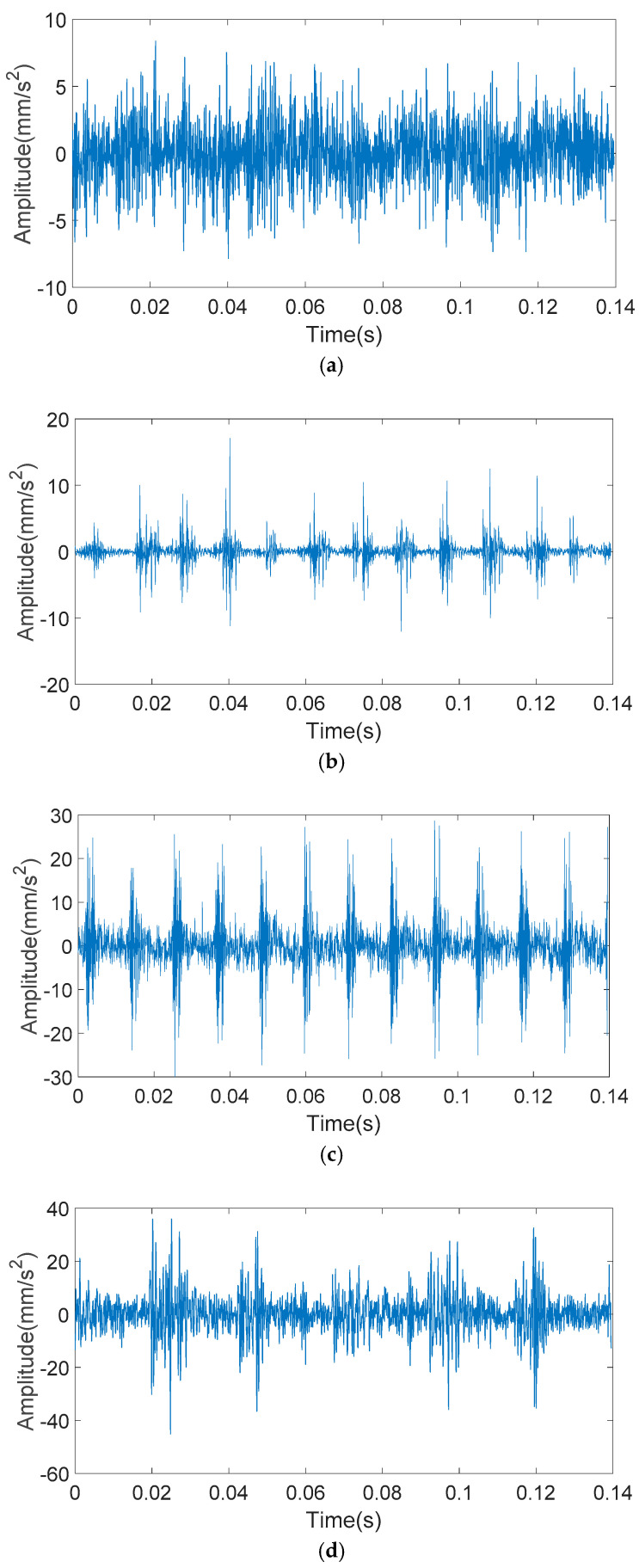
Outer race fault bearing vibration signals: (**a**) 0.5 mm of fault size; (**b**) 2 mm of fault size; (**c**) 3.5 mm of fault size; and (**d**) 5 mm of fault size.

**Figure 7:**
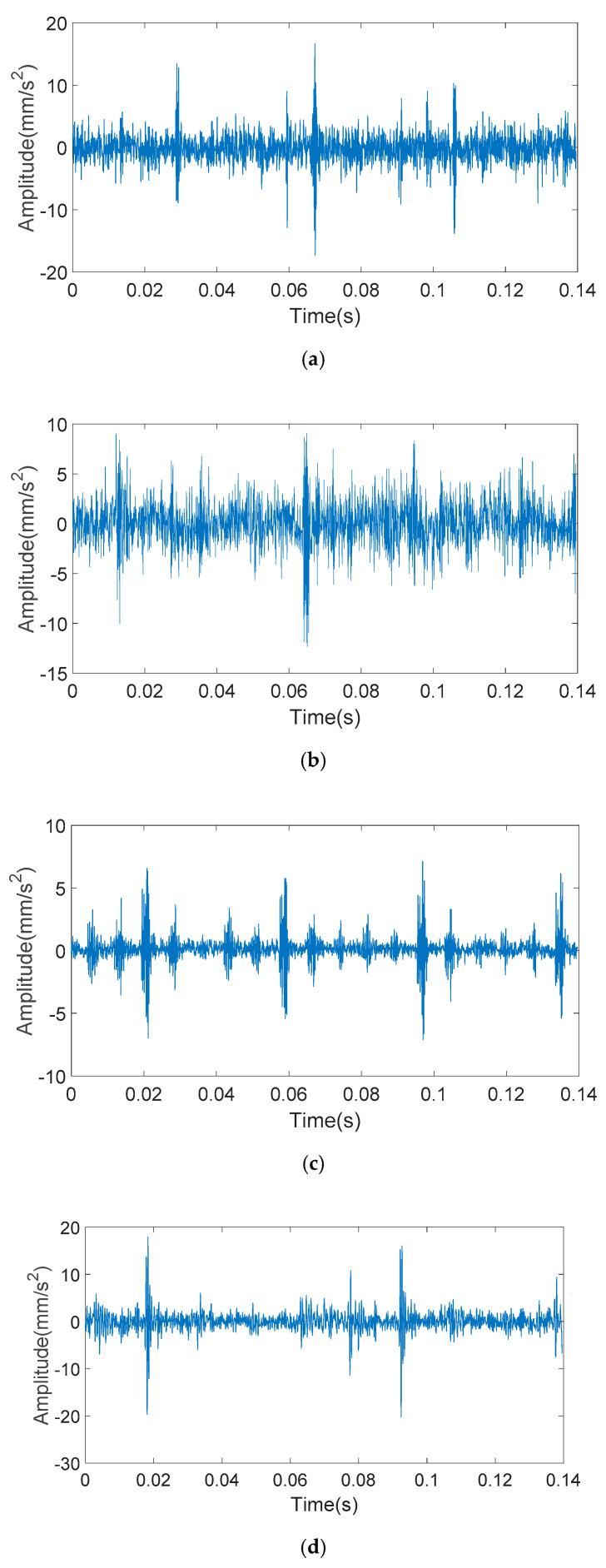
Inner race fault bearing vibration signals: (**a**) 0.5 mm of fault size; (**b**) 2 mm of fault size; (**c**) 3.5 mm of fault size; and (**d**) 5 mm of fault size.

The bearing vibration signals with inner and outer race faults of different fault sizes are shown in Figure 6 and Figure 7. It can be seen from Figure 6 that when the outer ring fault size is small, the impact of the vibration signal is masked by noise, where obvious impact cannot be seen. When the outer ring fault size is particularly large, the vibration signal will have multi-impact phenomenon. That is to say, the last impact has not yet ended and the next impact has emerged, which makes impacts overlap. It can be seen from Figure 7 that with the increase of inner ring fault size, the vibration signal will appear as a low frequency amplitude modulation phenomenon. However, when the inner ring fault size is too large, a higher amplitude impact will occur.

### 5.2. Establish QMM of Rolling Bearing

**Figure 8:**
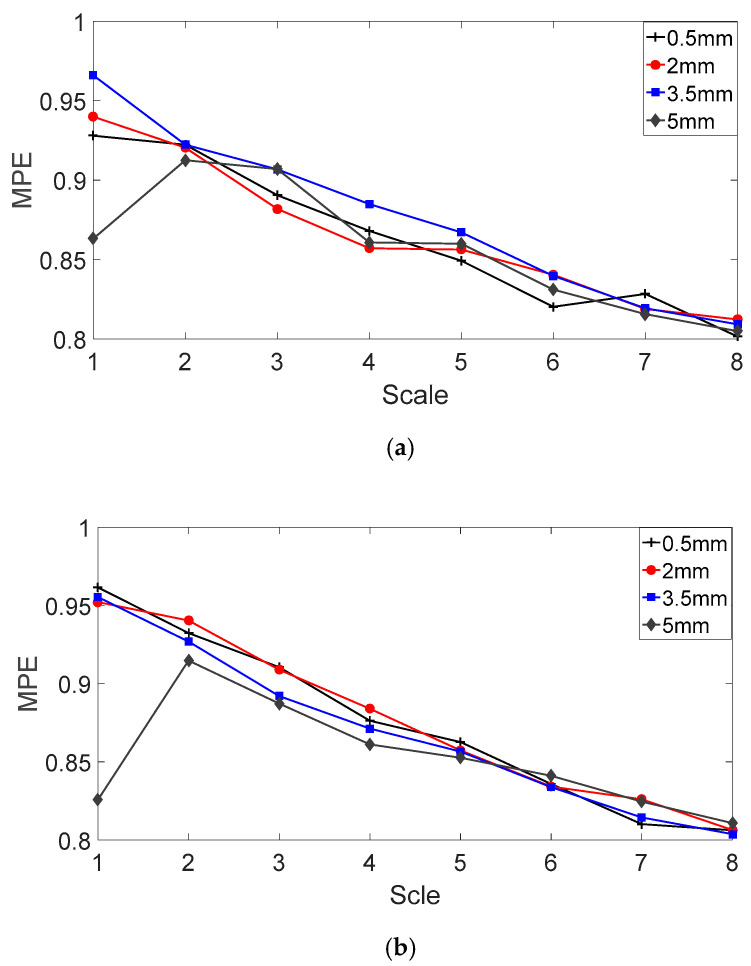
MPE of fault bearing vibration signals with fault sizes of 0.5 mm, 2 mm, 3.5 mm and 5 mm. (**a**) Fault bearing on outer ring; and (**b**) fault bearing on inner ring.

According to Reference [35], the embedding dimension *m* (Equation (12)) is 7, the time delay *λ* (Equation (12)) is 1 and the scale factor *s* (Equation (17)) is 8. After solving the multiscale permutation entropies (MPEs) of fault bearing vibration signals and the MPEs of fault bearing vibration signals with sizes of 0.5 mm, 2 mm, 3.5 mm and 5 mm, the results are shown in Figure 8. From Figure 8a,b, due to the existence of background noise and interference signals, the fault characteristics of the vibration signals are submerged, resulting in almost the same randomness and complexity for the vibration signals of different fault sizes. Therefore, this cannot be used to judge the relationship between MPE and fault sizes.

**Figure 9:**
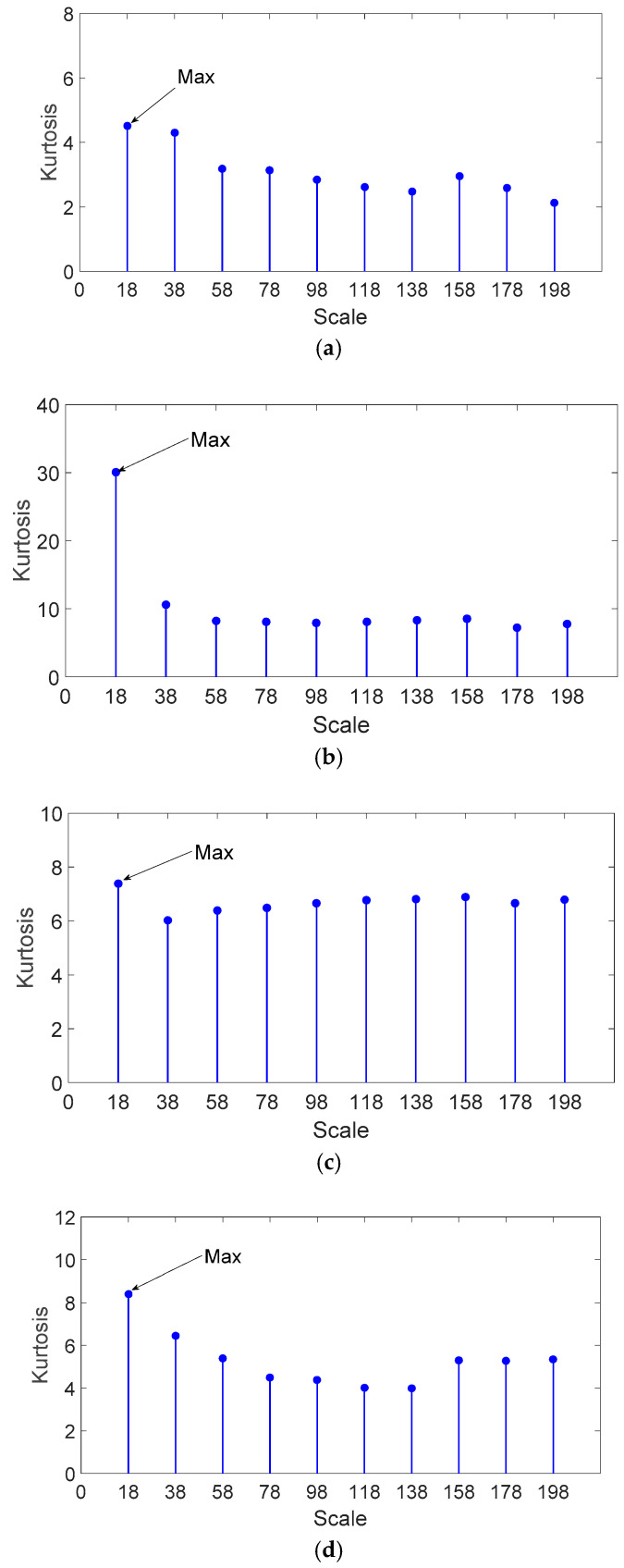
Kurtosis values of different scales for the analyzed signal of outer race fault bearing: (**a**) 0.5 mm of fault size; (**b**) 2 mm of fault size; (**c**) 3.5 mm of fault size; and (**d**) 5 mm of fault size.

In order to enhance the impulse characteristics and extract the time-domain geometric feature of fault bearing vibration signal, the vibration signals are morphologically analyzed (Equation (11)) with a flat structural element (SE). Raj et al. [34] proposed an optimal scale selection method of multiscale morphological filtering using the kurtosis principle (KS-MMF) and the method can be described as follows: The original vibration signal was first morphologically analyzed with SEs of 10 different scales spaced by 10% of the pulse duplicate period. After this, the kurtosis of the morphologically filtered signals was calculated. The filtered signal with the maximum kurtosis value is selected for further analysis. The outer race fault bearing vibration signals are analyzed through the KS-MMF method. According to the pulse repetition period T, the vibration signals are morphologically filtered with 10 flat SEs. Their kurtosis values are shown in Figure 9. It can be seen from Figure 9 that the scale corresponding to the maximum kurtosis is 18; therefore, 18 is the optimal scale for the outer race fault bearing vibration signals.

**Figure 10:**
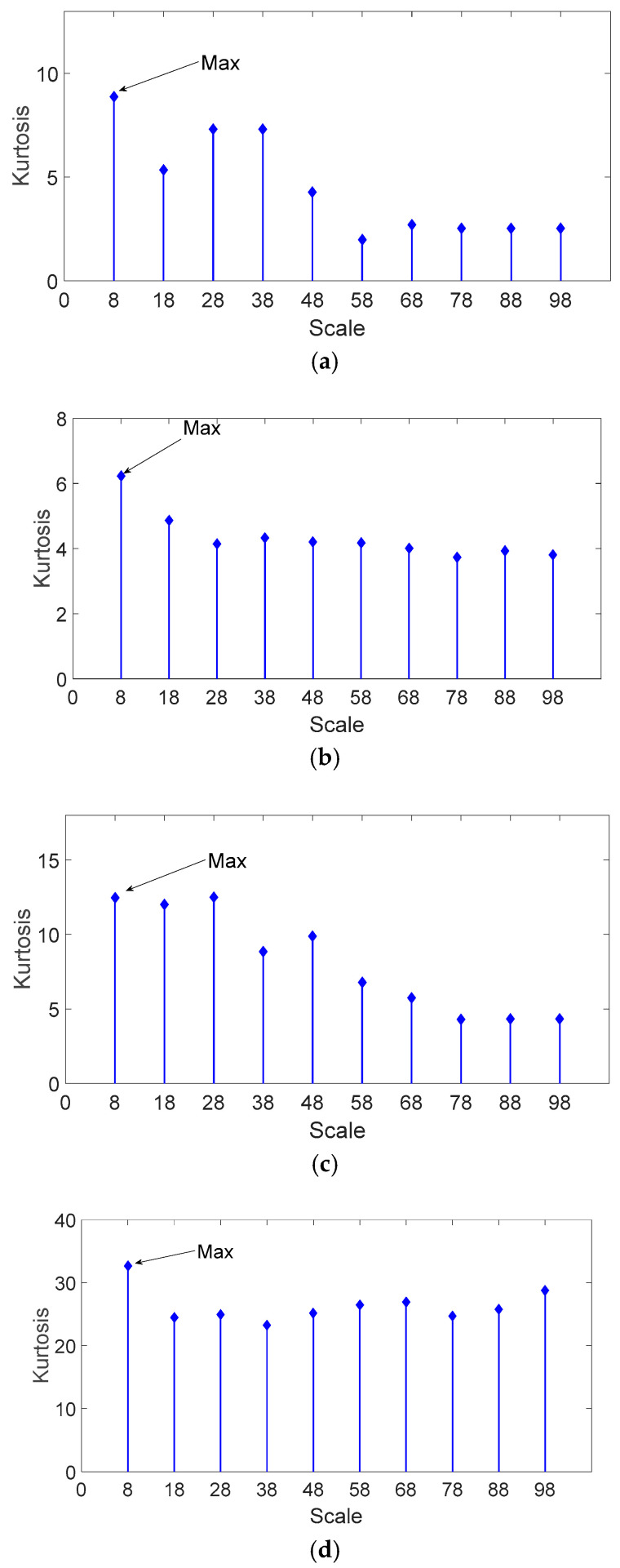
Kurtosis values of different scales for the analyzed signal of inner race fault bearing: (**a**) 0.5 mm of fault size; (**b**) 2 mm of fault size; (**c**) 3.5 mm of fault size; and (**d**) 5 mm of fault size.

The inner race fault bearing vibration signals are analyzed through the KS-MMF method. Their kurtosis values are shown in Figure 10. It can be seen from Figure 10 that the scale corresponding to the maximum kurtosis is 8; therefore, 8 is the optimal scale for the inner race fault bearing vibration signals.

**Figure 11:**
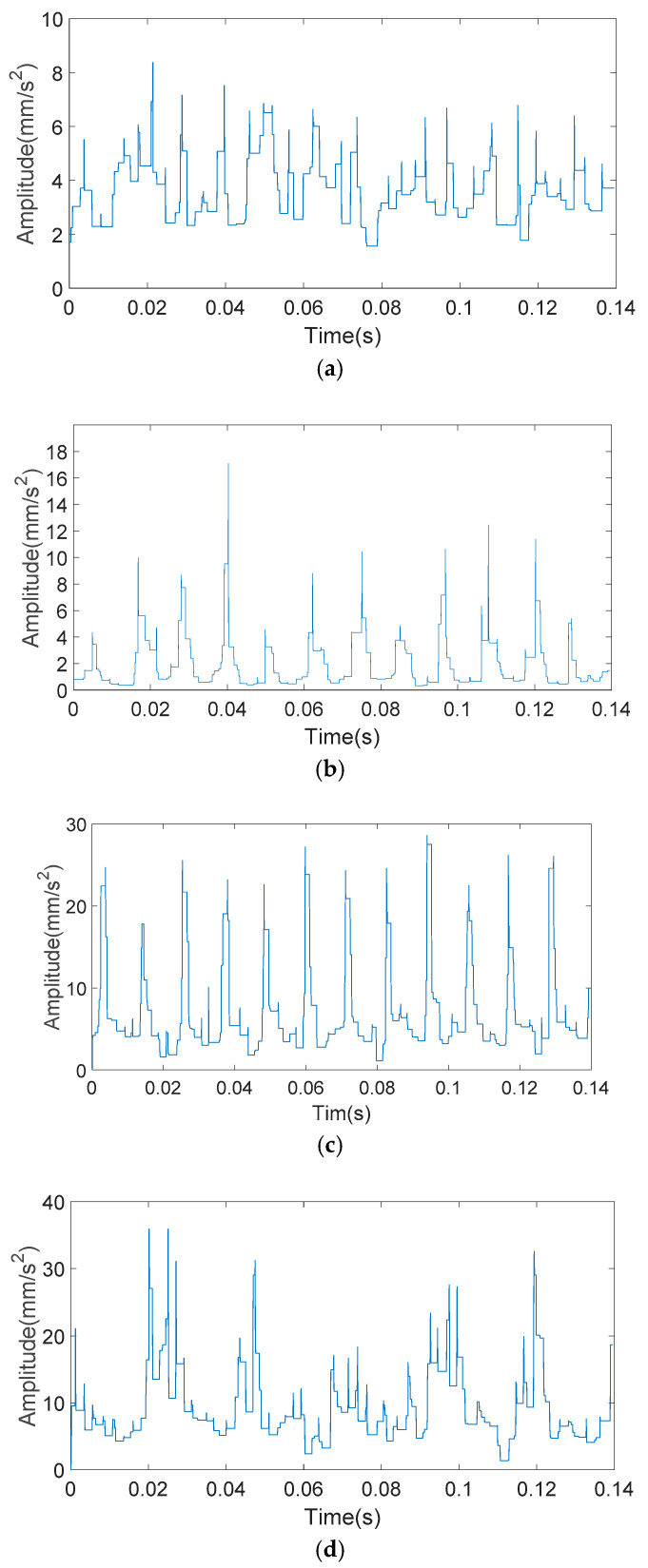
The analyzed vibration signals of outer ring fault: (**a**) 0.5 mm of fault size; (**b**) 2 mm of fault size; (**c**) 3.5 mm of fault size; and (**d**) 5 mm of fault size.

**Figure 12:**
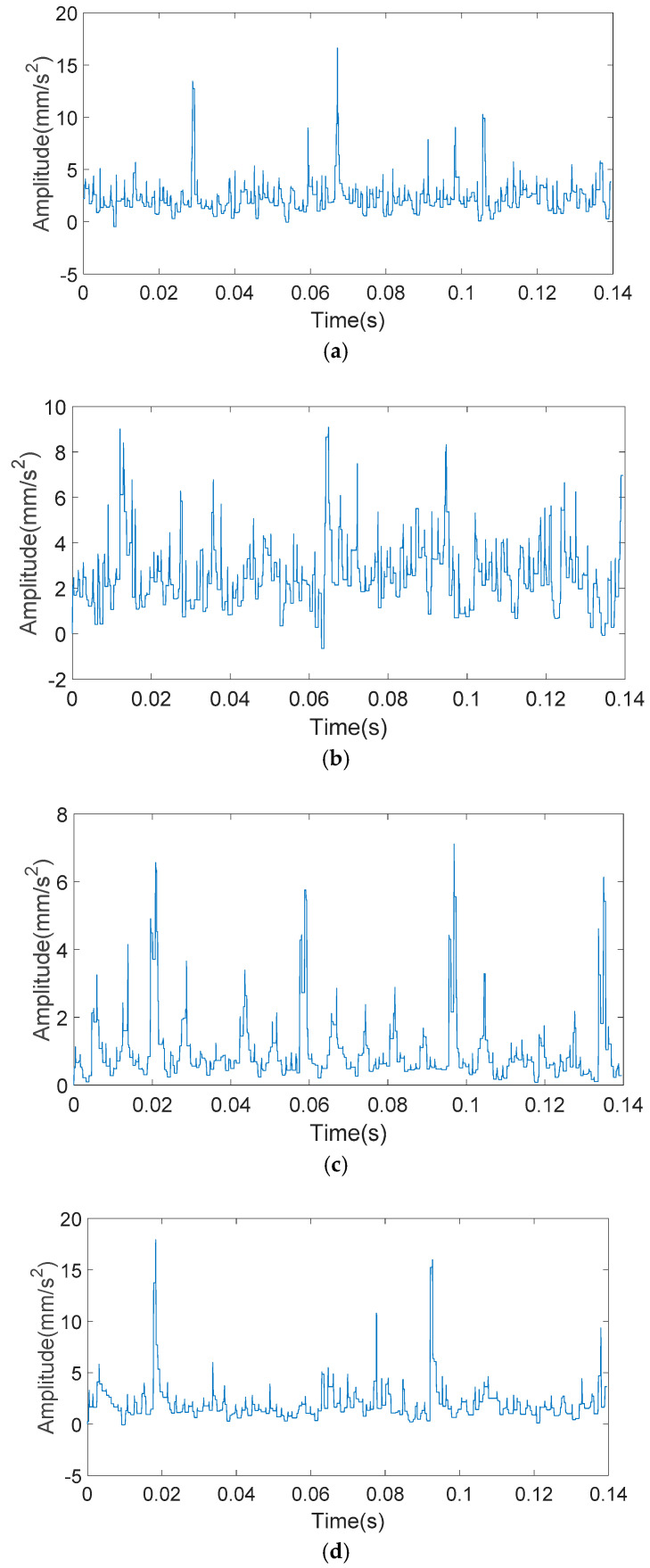
The analyzed vibration signals of inner ring fault: (**a**) 0.5 mm of fault size; (**b**) 2 mm of fault size; (**c**) 3.5 mm of fault size; and (**d**) 5 mm of fault size.

The analyzed signals through the KS-MMF method are shown in Figure 11 and Figure 12.

**Figure 13:**
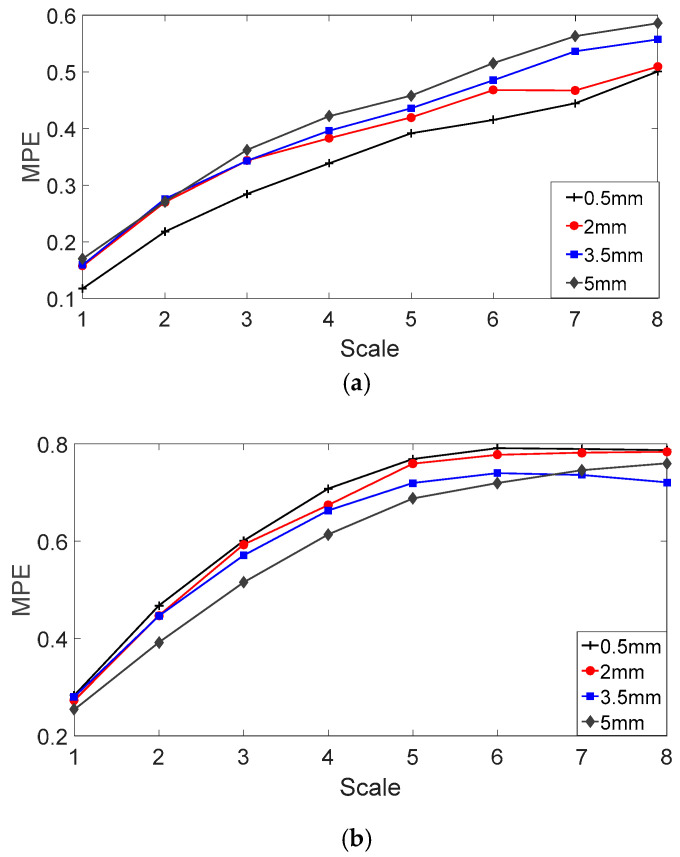
MPE of the analyzed vibration signals with different fault sizes: (**a**) Fault bearing on outer ring; and (**b**) fault bearing on inner ring.

The MPEs of the analyzed vibration signals are shown in Figure 13. Compared with MPEs of the original vibration signals, the MPEs of the analyzed vibration signals have a clear degree of discrimination regardless of the inner ring or outer ring fault. As the scale factor increases, the MPE value of the vibration signal becomes larger and tends to be flat.

**Figure 14:**
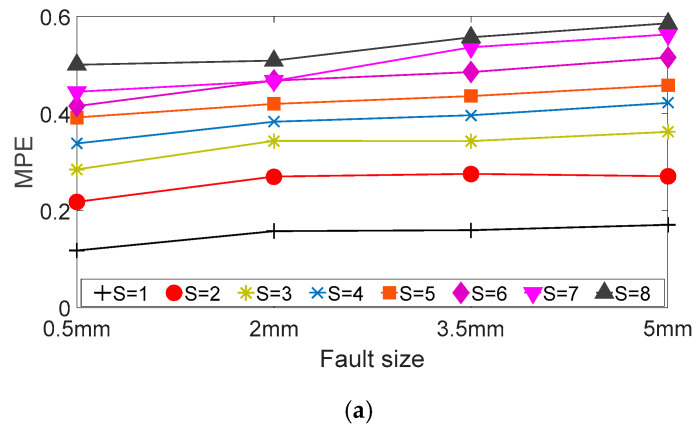

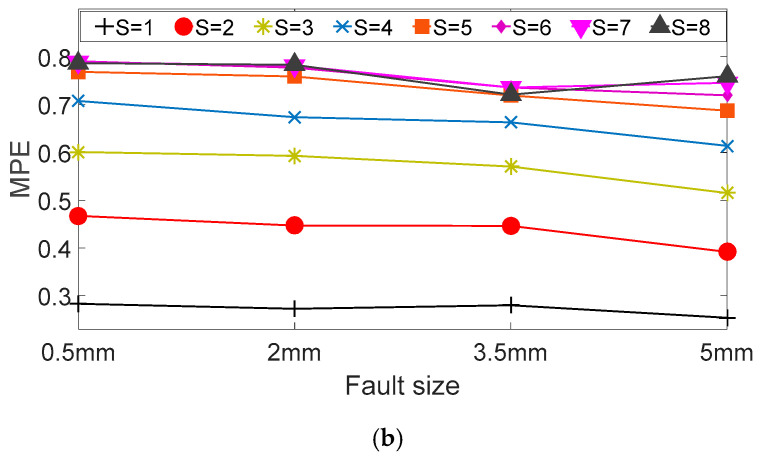
MPE of the analyzed vibration signals with different scale factors: (**a**) Fault bearing on outer ring; and (**b**) fault bearing on inner ring.

The MPE values of the vibration signal under different scale factors are shown in Figure 14. For the outer ring fault bearing signal in Figure 14a, an increase in the fault size results in the following changes: The MPE values under the different scale factors present an upward trend and the slopes of the different scale factors are different. For the inner ring fault bearing signal in Figure 14b, when the scale factor is less than 6, the MPE values of vibration signal present a downward trend after an increase in the fault size. However, when the scale factors are 6, 7 and 8, the MPE values with a fault size of 5 mm are larger than the MPE value with a fault size of 3.5 mm. In addition, the slope of MPE values under different scale factors is different.

**Figure 15:**
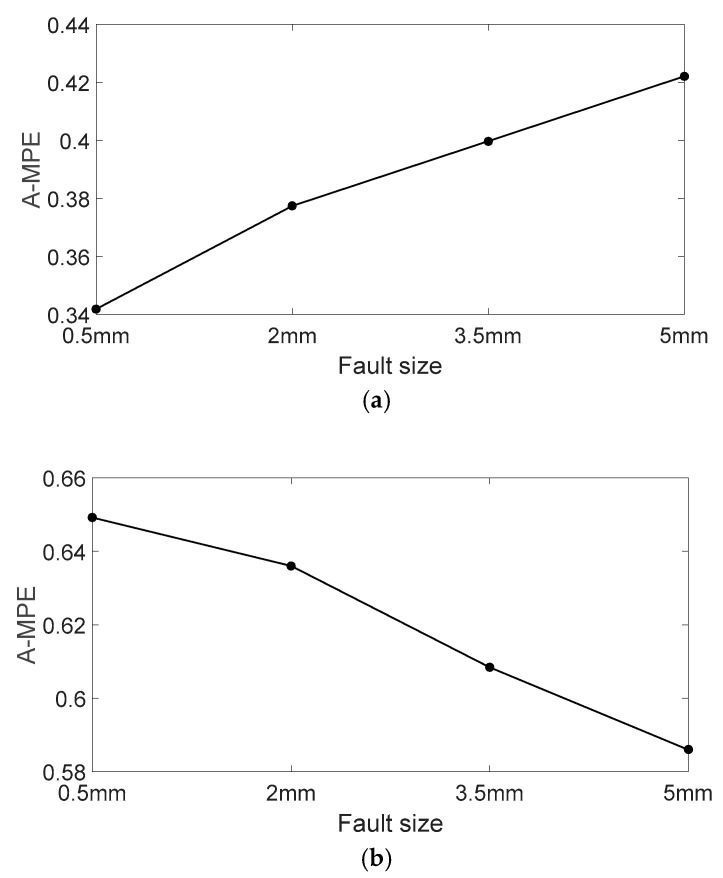
A-MPE of the analyzed vibration signals: (**a**) Fault bearing on outer ring; and (**b**) fault bearing on inner ring.

**Figure 16:**
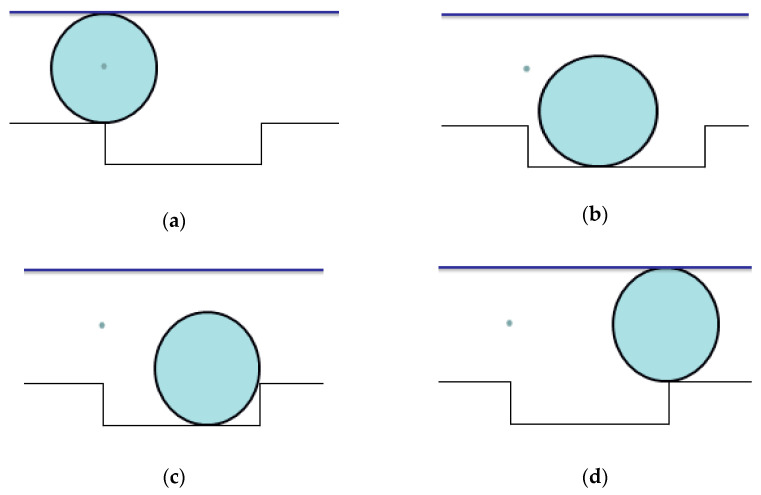
Schematic diagram of rolling element passing through the fault location: (**a**) when the rolling element just gets in touch with the fault edge; (**b**) when the rolling element just gets in touch with the bottom of the fault; (**c**) when the rolling element just gets in touch with the end of the fault; and (**d**) when the same rolling element leaves the other edge of the defect.

**Figure 17:**
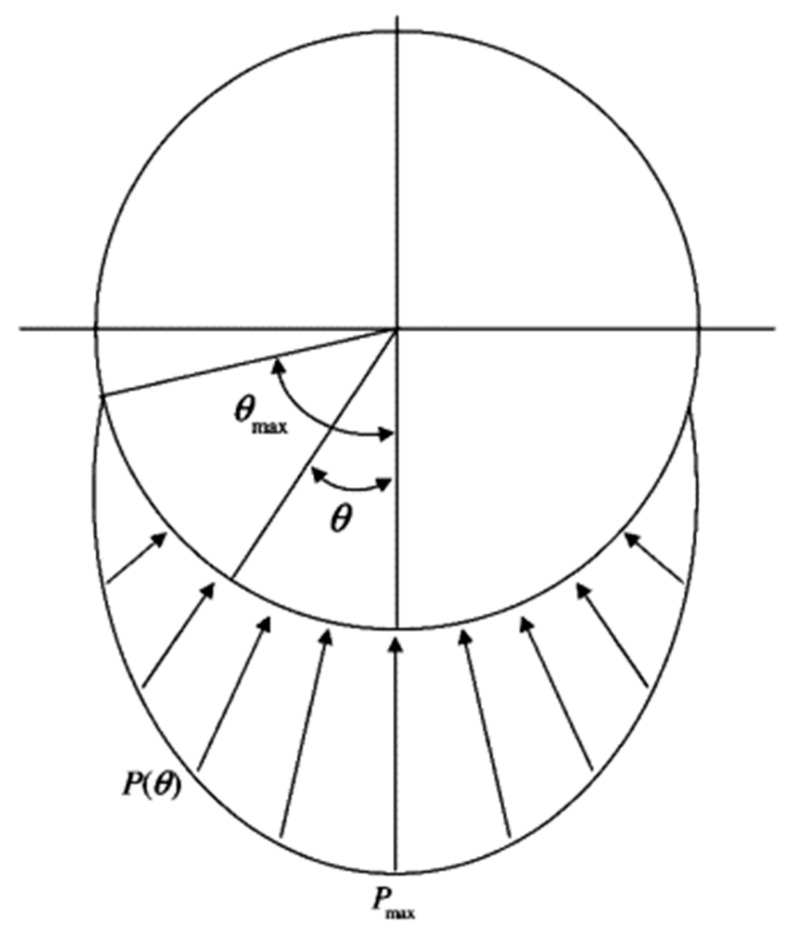
Load distribution in a bearing under radial load.

In order to reduce the impact of the scale on the MPE values, the average MPE (A-MPE) is solved in this paper. Figure 15 shows the A-MPE values of the vibration signals with different fault sizes. From Figure 15a, A-MPE value gradually increases with an increase in the outer ring fault size. When the fault point appears in the outer ring of the rolling bearing, the actual contact area between the rolling element and the outer raceway will decrease, which leads to the contact pressure first increasing before decreasing. In this way, a change in contact pressure leads to strong frequency modulation of the vibration signals. With an increase in outer ring fault size, the change of contact pressure increases gradually and the modulation phenomenon becomes increasingly obvious. Besides, in the case of a slightly larger bearing fault, the rising edge of the pulse is generated when the rolling element just gets in touch with the fault edge (Figure 16a), and the falling edge of the pulse is generated when the same rolling element leaves the other edge of the defect (Figure 16d); double-impact phenomenon is observed. In the case of a very larger bearing fault, the pulse is generated when the rolling element just gets in touch with the bottom of the fault (Figure 16c); multi-impact phenomenon can be observed. The schematic diagram of the rolling element passing through the fault location is shown in Figure 16. Therefore, the complexity of the vibration signal is getting larger and the signal is becoming more random [36]. Therefore, the vibration signal of bearing with a large outer ring fault size tends to have a higher A-MPE value, which is consistent with the theory. Based on the above analysis, as the outer ring fault increases, the A-MPE values become a rising trend with a slope greater than 0. From Figure 15b, the A-MPE gradually decreases with an increase in the inner ring fault size. When the fault point appears in the inner ring, the fault bearing vibration signal will have a low-frequency amplitude modulation [37,38]. With an increase in the inner ring fault size, the actual contact area between the rolling element and the inner raceway gradually decreases, which leads to increasingly significant amplitude modulation. Moreover, as shown in Figure 17, the radial load exerted on the rotating shaft causes an uneven load distribution around the inner ring of the bearing. Uneven load distribution causes low-frequency amplitude modulation of vibration signals, and the amplitude depends on maximum contact pressure *P_max_* [6]. With the increase of the inner ring fault size, the vibration signal appears as an obvious phenomenon of low-frequency oscillation, which decreases the randomness and complexity of the vibration signal and increases the orderliness. Therefore, the A-MPE shows a downward trend, which is also consistent with the theory. Based on the above analysis, as the inner ring fault increases, the A-MPE values become a decreasing trend with a slope of less than 0. In other words, when the fault locations of the inner and outer rings are different, the slope and trend of the A-MPE values are also different.

**Figure 18:**
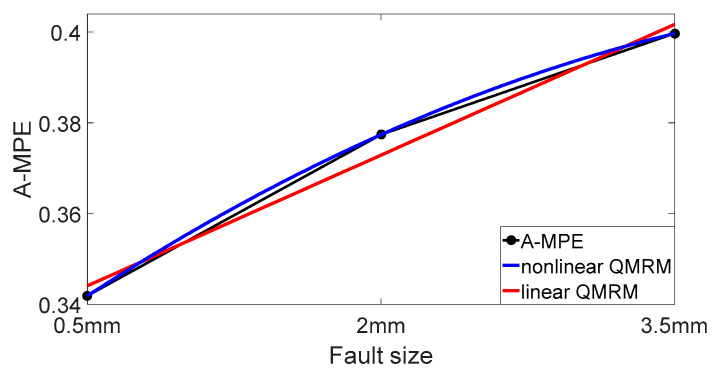
Linear and nonlinear QMMs of outer ring fault bearing.

**Figure 19:**
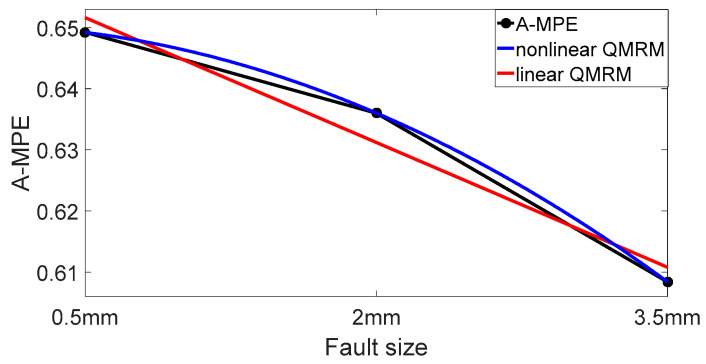
Linear and nonlinear QMMs of inner ring fault bearing.

The QMM is established by the bearing vibration signals with the fault sizes of 0.5 mm, 2 mm and 3.5 mm. According to the regression model, linear and nonlinear QMMs based on the rolling bearing fault size and A-MPE of the vibration signal are fitted. Linear and nonlinear QMMs of the outer ring fault bearing are shown in Figure 18, while the QMMs of the inner ring fault bearing are shown in Figure 19.

The QMM expression of the bearing vibration signal with an outer ring fault is shown in Table 3, where *x* is the fault size in mm. The error rate of the QMM is verified by A-MPE_5mm_, where A-MPE_5mm_ is A-MPE of the vibration signal with the fault size of 5 mm. The expression of the error rate is as follows:

(28) $$\delta =\frac{\left|S_{pre}-S_{act}\right|}{S_{act}}\times 100\%$$

where *S_pre_* is the fault size of prediction and *S_act_* is the actual fault size.

From Table 3, the actual fault dimension of the bearing outer ring is 5 mm. The predicted fault size is 4.599 mm according to linear QMM with an error rate of 8%. The vertex coordinates of the nonlinear QMM is (3.5, A-MPE_3.5mm_). The graph of the nonlinear QMM is convex and appears to have a downward trend after A-MPE_3.5mm_. Therefore, the nonlinear QMM does not predict the fault size. Therefore, the final QMM of the outer ring fault bearing in this paper is set to be linear, while the *A-MPE* = 0.0192*x* + 0.3339 is used as the best QMM.

The QMM expression of the bearing vibration signal with an inner ring fault is shown in Table 4. From Table 4, the real fault dimension of the bearing inner ring is 5 mm. The predicted fault size is 5.294 mm according to linear QMM with an error rate of 5.88%. The predicted fault size is 4.366 mm according to nonlinear QMM with an error rate of 12.68%. Therefore, the final QMM of the inner ring fault bearing in this paper is set to be linear and *A-MPE* = −0.0136*x* + 0.6584 is used as QMM.

Moreover, it can be deduced that if the A-MPE of a series of processed fault bearing signals has an upward trend, the signals are the inner ring fault signals. If the A-MPE of the processed fault signals have a downward trend, the signals are the inner ring fault signals. In other words, if the slope of the linear QMM of the vibration signal is greater than 0, the group vibration signals are the outer ring fault bearing vibration signals. Otherwise, it is the inner ring. Therefore, the localization diagnosis of the rolling bearing is realized.

## 6. Conclusions

According to the relationship between the multiscale permutation entropy (MPE) and the fault dimension of the rolling bearings, a novel quantitative and localization fault diagnosis method of rolling bearing is proposed based on the quantitative mapping model (QMM). The specific conclusions are as follows:

(1) With an increase in the outer ring fault size, the average multiscale permutation entropy (A-MPE) of the vibration signal gradually increases. With an increase in the inner ring fault size, A-MPE of the vibration signal gradually decreases.
(2) The experimental vibration signals are often accompanied by noise and interference signals, which affects the complexity of the vibration signals. In this paper, in order to extract the time-domain geometric feature of fault bearing vibration signal, the multiscale morphological filtering (MMF) method is used to analyze the vibration signal of fault rolling bearings and the A-MPEs of the analyzed vibration signals with different fault sizes have a clear degree of discrimination, which increases the accuracy of the QMM for fault prediction.
(3) The linear and nonlinear QMMs are used to predict the fault dimension by mapping the relationships respectively. When predicting both inner or outer ring faults, the results show that the accuracy of the linear QMM is higher than the nonlinear QMM, before the linear QMM is used as the final QMM.
(4) According to the slope of the linear QMM, the localization diagnosis of the rolling bearing is realized. If the slope of the linear QMM of the vibration signal is greater than 0, the group vibration signal is the outer ring fault bearing vibration signal. Otherwise, it is the inner ring fault.

The experiment data used in this paper are not adequate with a large span of the fault size, which affects the accuracy of the established QMM. After that, we will continue to study the quantitative analysis of rolling bearings and conduct further data analysis which improves the accuracy of QMM. In addition, each experimental setup has a specific QMM. Because of the different load, rotation speed and external conditions, the complexity of vibration signal is different. If the QMM is applied to the actual project, we can measure the vibration signals of the rolling bearings with different fault sizes, and establish its QMM to realize the fault prediction.

## Figures and Tables

**Table 1:** Filtering effects of morphological operators for the positive and negative impact.

Morphological Operators	Positive Impact	Negative Impact
Erosion operator	Inhibition	Smoothness
Dilation operator	Smoothness	Inhibition
Opening operator	Smoothness	Retention
Closing operator	Retention	Smoothness
Top-hat operator	Retention	Smoothness
Top-hat’s dual operator	Retention	Smoothness
Gradient operator	Smoothness	Smoothness
Difference operator	Retention	Retention

**Table 2:** Values of flat SE.

Scale	Length	Height
1	3	{0, 0, 0}
2	4	{0, 0, 0, 0}
*n*	*n* + 2	{0, 0, ..., 0, 0}

**Table 3:** QMM expression of bearing vibration signals with an outer ring fault.

Type	QMM	Actual Size	Prediction	Error Rate
Linear	*A-MPE* = 0.0192*x* + 0.3339	5 mm	4.599 mm	8%
Nonlinear	*A-MPE* = −0.0029*x*^2^ + 0.031*x* + 0.3271	5 mm	*no*	*no*

**Table 4:** QMM expression of bearing vibration signals with inner ring fault.

Type	QMM	Actual Size	Prediction	Error Rate
Linear	*A-MPE* = −0.0136*x* + 0.6584	5 mm	5.294 mm	5.88%
Nonlinear	*A-MPE* = −0.0032*x*^2^ – 0.0008*x* + 0.6504	5 mm	4.366 mm	12.68%
